# RNA Contaminates Glycosaminoglycans Extracted from Cells and Tissues

**DOI:** 10.1371/journal.pone.0167336

**Published:** 2016-11-29

**Authors:** Jasper J. van Gemst, Markus A. Loeven, Mark J. J. de Graaf, Jo H. M. Berden, Ton J. Rabelink, Cornelis H. Smit, Johan van der Vlag

**Affiliations:** 1 Department of Nephrology, Radboud Institute for Molecular Life Sciences, Radboud university medical center, Nijmegen, The Netherlands; 2 Department of Nephrology and Einthoven Laboratory for Vascular Medicine, Leiden University Medical Center, Leiden, The Netherlands; University of Patras, GREECE

## Abstract

Glycosaminoglycans (GAGs) are linear negatively charged polysaccharides and important components of extracellular matrices and cell surface glycan layers such as the endothelial glycocalyx. The GAG family includes sulfated heparin, heparan sulfate (HS), dermatan sulfate (DS), chondroitin sulfate (CS), keratan sulfate, and non-sulfated hyaluronan. Because relative expression of GAGs is dependent on cell-type and niche, isolating GAGs from cell cultures and tissues may provide insight into cell- and tissue-specific GAG structure and functions. In our objective to obtain structural information about the GAGs expressed on a specialized mouse glomerular endothelial cell culture (mGEnC-1) we adapted a recently published GAG isolation protocol, based on cell lysis, proteinase K and DNase I digestion. Analysis of the GAGs contributing to the mGEnC-1 glycocalyx indicated a large HS and a minor CS content on barium acetate gel. However, isolated GAGs appeared resistant to enzymatic digestion by heparinases. We found that these GAG extracts were heavily contaminated with RNA, which co-migrated with HS in barium acetate gel electrophoresis and interfered with 1,9-dimethylmethylene blue (DMMB) assays, resulting in an overestimation of GAG yields. We hypothesized that RNA may be contaminating GAG extracts from other cell cultures and possibly tissue, and therefore investigated potential RNA contaminations in GAG extracts from two additional cell lines, human umbilical vein endothelial cells and retinal pigmental epithelial cells, and mouse kidney, liver, spleen and heart tissue. GAG extracts from all examined cell lines and tissues contained varying amounts of contaminating RNA, which interfered with GAG quantification using DMMB assays and characterization of GAGs by barium acetate gel electrophoresis. We therefore recommend routinely evaluating the RNA content of GAG extracts and propose a robust protocol for GAG isolation that includes an RNA digestion step.

## Introduction

Glycosaminoglycans (GAGs) are linear, negatively charged polysaccharides and prominent components of extracellular matrices and cell surface glycan layers. GAGs are synthesized from repeating disaccharide building blocks and most GAGs, including heparan sulfate (HS), heparin, keratan sulfate, chondroitin sulfate (CS) and dermatan sulfate (DS), can be modified by sulfation, which renders them strongly negatively charged. For example, HS consists of N-acetylglucosamine and uronic acid disaccharide building blocks and can be sulfated at the N-, 2O-, 3O- and 6O-positions of the carbohydrate ring structures. The sequence of modifications along the carbohydrate backbone allows sulfated GAGs, particularly HS, to bind growth factors, chemokines and cellular adhesion molecules, such as fibroblast growth factors, interleukin-8, selectins and the macrophage-1 antigen (Mac-1), thereby regulating various physiological processes including cell growth, morphogenesis, coagulation and inflammation [1–9].

GAG expression and modifications are often tissue- and cell type-specific [10, 11]. Therefore, isolation and characterization of GAGs from different tissues or cell cultures is important to unravel tissue- and cell type-specific GAG structure and function [12]. Previously, we have isolated and characterized a unique mouse glomerular endothelial cell line (mGEnC-1) [13] and identified specifically sulfated HS domains in the glomerular endothelial glycocalyx that mediate chemokine binding and leukocyte trafficking during inflammation *in vitro* and *in vivo* [13–18]. Since the presence of many additional functional GAG domains in the glomerular endothelial glycocalyx is presumed, isolation and analysis of intact GAGs, e.g. using mass spectrometry, may yield novel structural information about functional GAG domains.

Described GAG extraction protocols usually involve release of GAGs using chaotropic buffers, non-ionic detergents, protease treatment or alkaline β-elimination, followed by removal of contaminants by enzymatic or chemical digestion, selective precipitation or chromatography [12, 19–21]. GAG quantification in mGEnC-1 GAG extracts initially suggested high yields, with HS as the major component of the mGEnC-1 glycocalyx, as was previously described [22]. However, the obtained HS fraction appeared largely resistant to digestion with bacterial heparinases I, II and III, suggesting that the sugars which co-migrated with HS standards during barium acetate agarose gel electrophoresis contained non-HS compounds. Subsequently we identified RNA as a major contaminant. Here, we describe a GAG isolation protocol including an RNAse treatment that yields GAG extracts that can be reliably visualized by agarose gel electrophoresis and quantified by the DMMB method.

## Materials and methods

### Cell culture and animal tissue

Conditionally immortalized mouse glomerular endothelial cells (mGEnC-1) were cultured as previously described [13]. Briefly, mGEnC-1 were grown at the proliferative temperature of 33°C in 1% gelatin (Sigma-Aldrich)-coated culture flasks (Corning Life Sciences) with DMEM/Ham’s F12 medium (3:1; Life Technologies) supplemented with 5% fetal bovine serum (FBS; Bodinco), 1% penicillin/streptomycin (PS; Life Technologies) and 20 units (U)/ml recombinant mouse interferon-γ (IFN-γ; PeproTech). For differentiation, mGEnC-1 were seeded at 25% density in uncoated culture flasks and cultured for 7 days in DMEM/Ham’s F12 without IFN-γ at the non-permissive temperature of 37°C. Primary human umbilical vein endothelial cells (HUVEC) were grown to confluence on 1 μg/cm^2^ bovine fibronectin (Bio-Connect)-coated culture flasks in endothelial cell growth medium (EGM)-2 (Lonza). Immortalized retinal pigmental epithelial cells (ARPE-19) were grown to confluence in culture flasks in DMEM/Ham’s F12 (1:1) supplemented with 10% FBS and 1% PS. All cell lines were maintained in T75 culture flasks in a 5% CO_2_ humidified environment at 37°C.

Mouse organs were collected from wild-type C57BL/6J mice sacrificed by cervical dislocation. Animal experiments were approved by the animal ethics committee of the Radboud University.

### Isolation of GAGs from cells and tissues

Original protocol [23, 24]: Cell monolayers were thoroughly washed with phosphate-buffered saline (PBS), and digested overnight at 37°C with 125 μg/ml proteinase K (Merck Millipore) in 2 ml extraction buffer (50 mM Tris-HCl, 10 mM NaCl, 3 mM MgCl_2_ and 1% triton X-100, pH7.9) per 75 cm^2^ confluent cell monolayer. The lysate was recovered from the culture flask and heated to 95°C for 10 min to deactivate proteinase K before adding 7.5 U/ml DNase-I (Qiagen) and incubating overnight at 37°C. The digested lysate was then mixed 1:1 with 4 M sodium chloride to dissociate GAG-bound peptides, followed by mixing 1:1 with chloroform and centrifugation for 30 min at 4500xg. The top (aqueous) layer containing purified GAGs was collected and dialyzed thoroughly against 18.2 MΩ.cm deionized water (MQ). GAG solutions were then dried using a SC200 Speed Vac centrifugal evaporator (Savant Instruments). Before analysis, GAG preparations were reconstituted in MQ. GAGs from cryosections of C57BL/6J mouse tissues (i.e. heart, liver, spleen and kidney) were isolated by the same protocol described above.

Adapted protocol: An excess of RNase-I (10 U/ml; Thermo Scientific) was added to the DNase mixture for subsequent extractions. Where indicated, GAG extracts were treated with a mixture of 0.25 U/ml heparinase I, II and III (Sigma).

### DMMB analysis of GAGs

DMMB solution was prepared as previously described [23, 25] with minor modifications. Fifty mg DMMB (Sigma) was dissolved in 25 ml ethanol, filtered through Whatman filter paper and used to prepare a solution containing a final concentration of 0.1 mg/ml DMMB, 5% v/v ethanol, 0.2 M guanidine hydrochloride (GuHCl), 0.2% w/v sodium formate and 0.2% w/v formic acid. Subsequently, the dye mixture was diluted (1:1) with an identically prepared, DMMB-free buffer to create a stable solution. One ml of the prepared DMMB solution was added to 100 μL of sample and vortexed for 30 minutes. The GAG:DMMB complex was precipitated by centrifugation at 10.000xg for 10 minutes and the supernatant was aspirated carefully. The precipitate was then reconstituted by vortexing for 30 minutes in 250 μL decomplexant solution (4 M GuHCl, 10% 1-propanol and 50 mM sodium acetate, pH6.8). Two hundred μL of dissolved GAG:DMMB mixture was transferred to a 96 wells plate and the absorbance at 650 nm was measured using a Bio-Rad Multiplate Reader (Bio-Rad). To quantify the GAG concentration, absorbances were compared to different amounts (0 to 40 μg/ml) of heparan sulfate from bovine kidney as a standard (HSBK; Sigma). GAG concentrations are given as mean ± s.e.m. Significance was determined by ANOVA.

### Barium acetate agarose gel electrophoresis and silverstaining of GAGs

Analysis of obtained GAGs on agarose gel was performed as described previously [26] with minor modifications. In short, 500 mg multipurpose agarose (1%; Roche) was dissolved by heating in 50 ml 50 mM barium acetate (electrophoresis buffer, pH 5.0), 13 ml were cast on the hydrophilic side of a 85 x 100 mm gelbond film (Lonza) placed on a glass slide and wells were excised once the gel had set. A mass of 0.5–1 μg of isolated GAGs were diluted 6x in electrophoresis buffer containing 20% glycerol and 0.01% bromophenol blue. Five μl/well GAG extract were loaded and electrophoresis was performed in electrophoresis buffer at 60V on a LKB bromma 2117 multiphor electrophoresis unit (LKB, Bromma, Sweden). Gels were stained and fixed overnight with 0.1% w/v Azure A (Sigma) in 50 mM sodium formate (pH 3.5) and 10 mM magnesium chloride, destained with 10 mM sodium acetate (pH 5.5) and air dried. Gels were washed twice for 10 minutes in 1% triton X-100 (Sigma) in MQ and washed again thoroughly with MQ to remove residual triton X-100. Silverstaining was performed by adding 100 ml freshly prepared silverstaining solution, consisting of a mixture of 60 mM NH_4_NO_3,_ 30 mM AgNO_3_, 3.5 mM tungstosilicic acid and 0.15 mM 37% formaldehyde, 0.47 M Na_2_CO_3_ (1:1, v/v). The reaction was stopped with 1% acetic acid in MQ and gels were air dried. GAG extracts were compared to commercial standards for HS, DS and CS (Sigma).

### Ethidium bromide agarose gel electrophoresis

RNA presence in 0.5–1 μg of isolated GAG extracts was analyzed on 1% agarose gel in Tris-Boric acid-EDTA (TBE; Invitrogen) with 0.01% ethidium bromide.

## Results and Discussion

**Fig 1:**
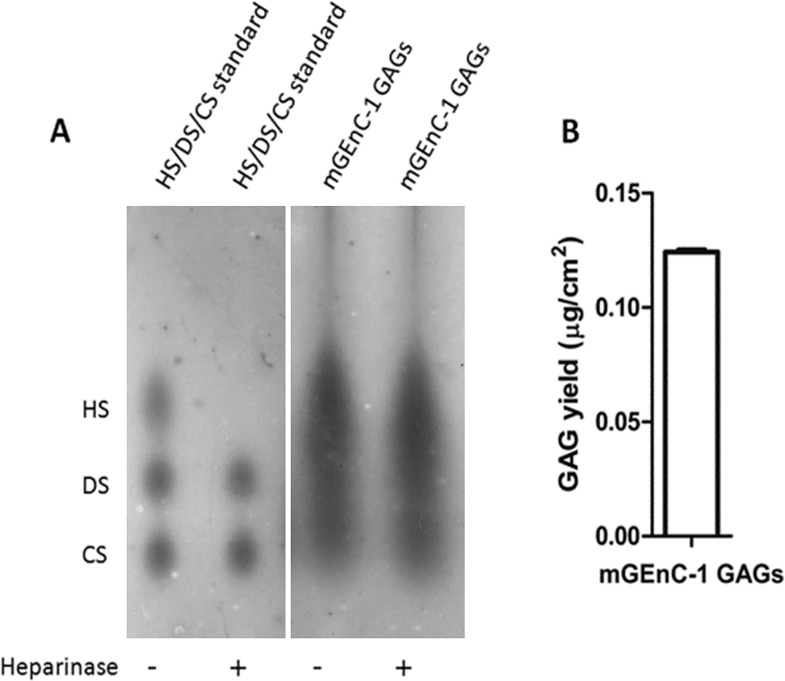
Characterization of mGEnC-1 GAGs by barium acetate gel electrophoresis and DMMB analysis. The GAG content in extracts from conditionally immortalized mouse glomerular endothelial cells (mGEnC-1) was visualized by barium acetate gel electrophoresis (A), and quantified relative to heparan sulfate from bovine kidney using 1,9-dimethylmethylene blue (B). Analysis of heparinase I, II and III-treated mGEnC-1 GAGs on gel indicated no degradation of the spot that co-migrates with the HS standard.

GAGs in the glomerular endothelial glycocalyx mediate important functions [13–18], therefore we aimed to isolate pure GAGs from cultured glomerular endothelium to obtain novel information on composition and structure. To isolate and characterize GAGs expressed by mGEnC-1, a published protocol for the isolation of GAGs from tissues [23, 24] was followed. The GAG composition of the mGEnC-1 glycocalyx as assessed by barium acetate agarose gel electrophoresis implied a large HS and smaller CS content (Fig 1A), which was in line with the previously described HS:CS ratio of 4:1 for endothelium [22]. Isolated GAGs were quantified by an adapted 1,9-dimethylmethylene blue (DMMB) GAG quantification assay based on the Farndale method [23–25], which relies on the formation of a GAG-cationic dye complex. GAGs obtained from mGEnC-1 were quantified relative to HSBK, indicating a yield of ~0.12 μg GAGs per cm^2^ cell monolayer (Fig 1B). To confirm the relative contribution of HS or CS to the mGEnC-1 glycocalyx, GAG preparations were treated with heparinases I, II and III to digest HS. However, the glycocalyx-derived GAG spot co-migrating with HS standards was not affected by enzymatic degradation with heparinases I, II and III (Fig 1A), suggesting a major impurity with an electrophoretic mobility comparable to HS.

**Fig 2:**
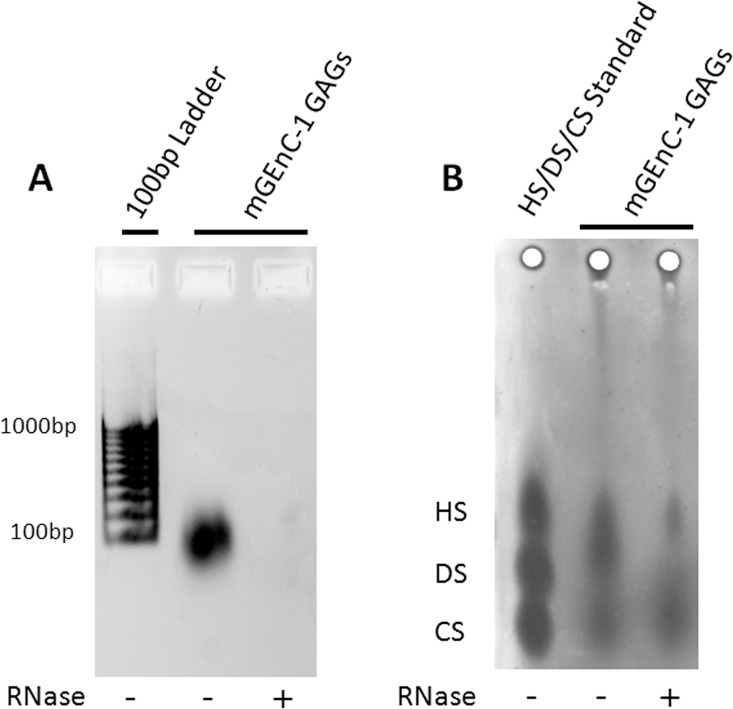
Characterization of mGEnC-1-derived GAGs reveals RNA as a major contaminant. The RNA content in extracts from conditionally immortalized mouse glomerular endothelial cells (mGEnC-1) was visualized by ethidium bromide agarose gel electrophoresis (A), and barium acetate gel electrophoresis (B) before and after RNase treatment. Enzymatic degradation of RNA in mGEnC-1 GAG extracts removes the RNA band observed on ethidium bromide gel, and a large spot that appears to co-migrate with HS on the barium acetate gel.

Therefore, the original isolation protocol was re-evaluated for potential sources of contamination. We reasoned that RNA may be contaminating the GAG extracts, since a high density of negative charges in RNA could result in co-purification and detection by cationic dyes. When the purity of GAG extracts was assessed on ethidium bromide agarose gels, a significant polynucleotide impurity was found, which could efficiently be removed by RNase-I digestion (Fig 2A). Furthermore, when the RNase-I-treated mGEnC-1 GAG extract was re-evaluated on barium acetate gels these GAG extracts showed a much fainter spot co-migrating with the HS standards (Fig 2B). Together, the ethidium bromide and barium acetate gel electrophoresis data suggest that, under the conditions applied, RNA is a major contaminant in the GAG extract obtained from mGEnC-1 cells. RNA digestion with RNase-I enables determination of the actual GAG composition and the relative contribution of HS and CS to the mGEnC-1 glycocalyx.

**Fig 3:**
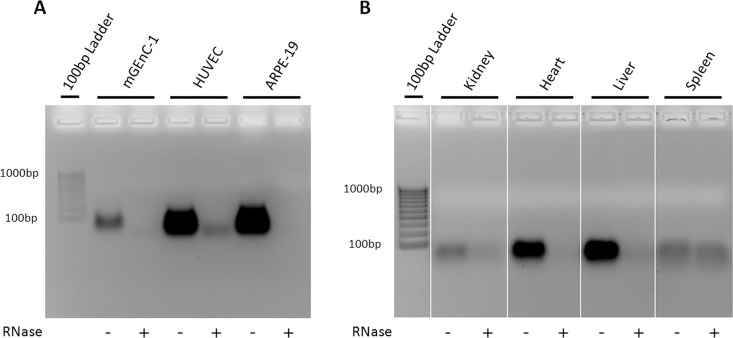
Glycosaminoglycans (GAG) extracts contain significant RNA impurities, which can be digested by RNase-I treatment. Visualizing nucleotide impurities in GAG preparations from conditionally immortalized mouse glomerular endothelial cells (mGEnC-1), human umbilical vein endothelial cells (HUVEC), immortalized retinal pigmental epithelial cells (ARPE-19) (A), and mouse kidney, heart, liver and spleen (B) on ethidium bromide agarose gels, revealed significant RNA contaminations. RNase-I treatment efficiently removed the contamination from mGEnC-1 and ARPE-19 GAGs, though minor impurities remained in HUVEC-, kidney- and spleen-derived extracts.

**Fig 4:**
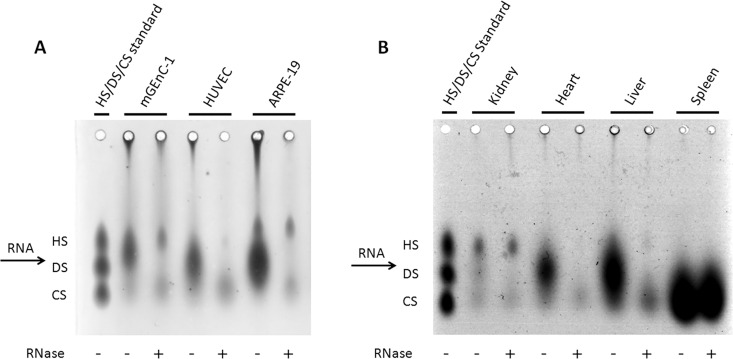
RNA impurities interfere with the analysis of glycosaminoglycans (GAGs) using barium acetate agarose gel electrophoresis. Resolving untreated GAG extracts by barium acetate gel electrophoresis suggested relatively large amounts of heparan sulfate (HS) and dermatan sulfate (DS) and relatively little chondroitin sulfate (CS) in GAGs obtained from mouse glomerular endothelial cells (mGEnC-1), human umbilical vein endothelial cells (HUVEC) and immortalized retinal pigmental epithelial cells (ARPE-19) (A). GAG extracts from mouse tissues appeared to contain large amounts of DS (heart and liver) and CS (spleen), whereas kidney-derived GAGs were enriched in HS, but also contained DS and CS (B). However, the observed staining patterns seemed to result from contaminating RNA co-migrating between HS and DS, as RNase-I treatment revealed the actual GAG spots corresponding to primarily HS and CS.

**Fig 5:**
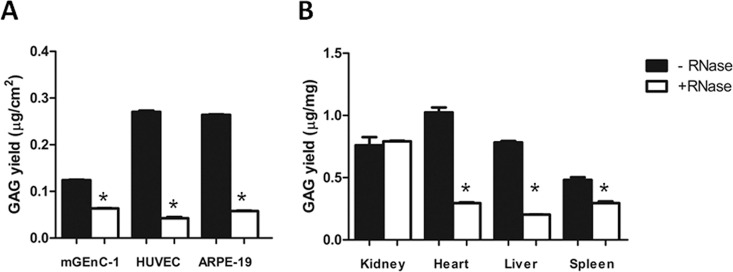
RNA contamination of glycosaminoglycan (GAG) extracts leads to a significant overestimation of GAG yields. The GAG content in extracts from conditionally immortalized mouse glomerular endothelial cells (mGEnC-1), human umbilical vein endothelial cells (HUVEC), immortalized retinal pigmental epithelial cells (ARPE-19) (A) and C57BL/6J mouse kidney, heart, liver and spleen (B) was quantified relative to heparan sulfate from bovine kidney using 1,9-dimethylmethylene blue. The apparent yield in untreated GAG samples from cell cultures was significantly overestimated 2- to 6-fold compared to RNase-treated samples, indicating that RNA contamination interferes with the charge-based DMMB quantification method. RNA also interfered with the quantification of GAGs obtained from heart, liver and spleen, but not in kidney cortex extracts. GAG concentrations are presented as μg/cm^2^ confluent cell monolayer or μg/mg wet tissue. Results are given in means ± s.e.m. *P<0.05 by Anova.

To determine whether RNA is a common contaminant in other cell- and tissue-derived GAG extracts, and whether this may affect further analysis, GAGs were isolated from HUVECs and ARPE-19 cells, and from mouse kidney, heart, liver and spleen, with and without including an RNase-I digestion step during purification. Like mGEnC-1 cells, both HUVEC- and ARPE-19-derived GAGs contained substantial RNA impurities that were detected in the ethidium bromide and barium acetate agarose gels (Figs 3A and 4A). Furthermore, analysis of GAG yield by DMMB assays suggested about 5-fold higher RNA concentrations in HUVEC and ARPE-19 GAG extracts compared to mGEnC-1 GAG extracts, whereas the true GAG concentration measured by DMMB after RNase treatment was comparable between 40 and 60 ng/cm^2^ confluent cell culture (Fig 5A). Kidney, heart, liver and spleen extracts all contained variable amounts of RNA impurities, some of which appeared less susceptible to RNase-I treatment, particularly in spleen extracts (Figs 3B and 4B). GAG isolation from some tissues, such as the spleen, may therefore require a higher RNase-I concentration for complete RNA degradation. In most tissues, except the kidneys, degradation of the RNA contaminant also resulted in a pronounced reduction in RNA/GAG yield as determined by DMMB assays (Fig 5B).

The susceptibility of GAG quantification using DMMB to contaminating polyanions has recently been discussed in the field of tissue engineering as well [27]. Studies on GAGs in synovial fluid showed that contaminating RNA and DNA at concentrations above 20 μg/ml result in the overestimation of GAG content [28]. Accordingly, quantification of cell/tissue-derived GAG extracts using DMMB assays in our study revealed a major reduction in apparent GAG yield from 120 to 280 ng before RNase treatment to between 40 to 60 ng of GAGs per square cm of cultured cells after RNase treatment (Fig 5A), and a GAG yield of 500 to 1000 ng before RNase treatment to 200 to 300 ng of GAG per mg of tissue after RNase treatment (Fig 5B). However, the constant yield of GAGs obtained from mouse kidney tissue, may indicate that the concentration of contaminating RNA is below the detection threshold for detection with DMMB, since analysis on gel did reveal an effect of RNase treatment (Fig 4B).

The results from our studies thus clearly demonstrate that GAGs extracted from both tissues and cell-lines contain varying amounts of RNA (Fig 3), which interfere with identification of the GAGs on barium acetate agarose gel electrophoresis (Fig 4), since RNA appeared to co-migrate in between the HS and DS fraction, and with the quantification of GAGs in DMMB assays (Fig 5). We showed that most of these RNA impurities could be efficiently removed by overnight incubation with an excess of RNase-I.

Since many of the more recent GAG extraction protocols do not include treatments to remove RNA contaminants, it was initially hypothesized that the inherent instability of RNA, as well as abundant endogenous/exogenous RNases would be sufficient to remove contaminating RNA. Many studies have focussed on isolation of GAGs for quantification or structural analysis, resulting in a large variety in protocols for GAG extraction. In some of the earliest publications describing GAG isolations, DNase and RNase were used to remove contaminating polynucleotides from the GAG extracts [29–31]. However, more recent publications no longer describe (a combination of) these endonucleases [23, 24, 32–38], which will result in the ample presence of RNA in GAG extracts. Of course, depending on the specific downstream application of the GAG extract and additional purification steps such as ion exchange chromatography, not all GAG extractions require RNA digestion [39–41]. For example, when incorporating radioactive sulfate into GAGs for the quantification of GAG synthesis in cultured cells, the presence of RNA in the GAG extract may be neglected [42–44]. But also in these settings RNA impurities can become highly relevant during functional assays, because of the similar physical characteristics between GAGs and RNA. Monitoring the RNA content of GAG extracts after ethidium bromide agarose gel electrophoresis or by measuring the absorbance at 260 nm during purification can reveal contamination, particularly when combined with high resolution techniques such as capillary electrophoresis [45]. Furthermore, several protocols use alkaline treatment to release GAGs into solution. RNA is highly susceptible to alkaline hydrolysis [46], and protocols including alkaline treatment are therefore less likely to contain significant RNA impurities [47–51]. A drawback of this method however is that both depolymerisation of GAGs and loss of functional groups, including sulfates, can occur during the incubation of GAGs in basic solutions [52, 53]. Alkaline treatment may therefore not be an ideal approach when the goal is to obtain more specific structural information about the GAGs expressed in tissues or on cultured cells.

Thus, the adaptation of a protocol for GAG extraction must be chosen carefully, since the application described in the original article may require a lesser degree of purity of the final GAG extract, as in our studies we experienced problems with the RNA impurity that is not described in the source of our GAG isolation protocol [23, 24]. In theory, this may lead to false detection of GAGs after barium acetate gel electrophoresis or DMMB analysis, e.g. when HS presence is concluded from azure A staining on agarose gels based on a “known” band position compared to DS and CS [54] and may therefore be wrongfully assigned. Since polynucleotides are often not considered as potential contaminants in GAG extracts, the complexation with cationic dyes that occurs in DMMB assays may result in overestimated GAG yields and wrong assignment of relative GAG compositions [23, 24, 32–38]. It is difficult to discern from the results of studies that lack controls for RNA content whether GAG extracts were contaminated by RNA. However, excessively high GAG yields and intense GAG spots on barium acetate agarose, which cannot be removed by digestion with GAG-specific glycosidases, can suggest a contamination with polynucleotides.

**Fig 6:**
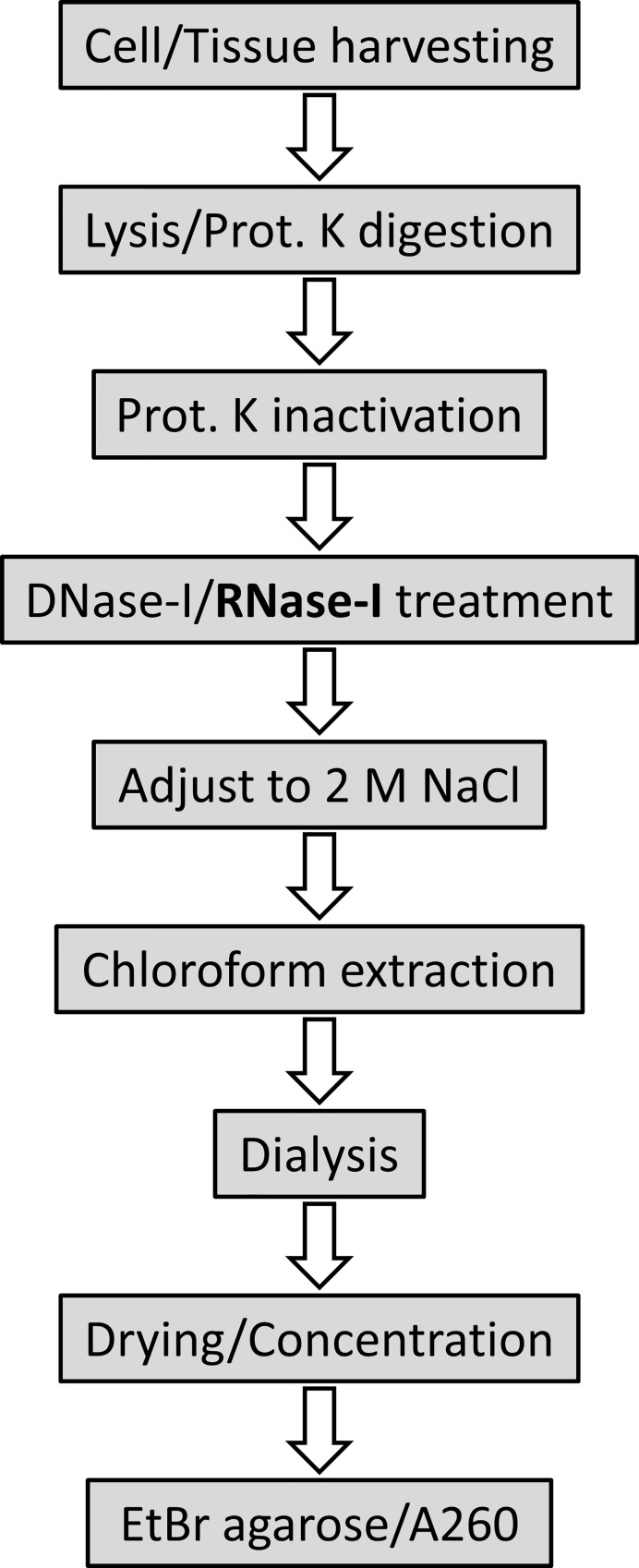
Schematic workflow for glycosaminoglycan (GAG) extraction including RNase treatment Cell/tissue lysates are treated overnight with proteinase K (Prot. K), followed by DNase-I and RNase-I treatment and finally chloroform extraction and dialysis to remove contaminating proteins/DNA/RNA. After drying/concentration of GAG extracts, the purity of the preparations is assessed using ethidium bromide (EtBr) agarose gel electrophoresis, or by measuring the absorbance at 260 nm (A260).

In conclusion, the similar physical characteristics of GAGs and nucleic acids can result in significant RNA contamination of GAG extracts, which interferes with GAG compositional analysis on agarose gels and charge-based quantification. We therefore recommend to routinely evaluate the RNA content of GAG extracts and propose a robust protocol for GAG isolation that includes an RNA digestion step (Fig 6).
